# Strategic directions for strengthening nursing and midwifery: potentialities and connections in the complex perspective*

**DOI:** 10.1590/1518-8345.4456.3380

**Published:** 2020-10-19

**Authors:** Ítalo Rodolfo Silva, Isabel Amélia Costa Mendes, Carla Aparecida Arena Ventura

**Affiliations:** 1 Universidade Federal do Rio de Janeiro, Campus Macaé, Macaé, RJ, Brazil.; 2 Universidade de São Paulo, Escola de Enfermagem de Ribeirão Preto, PAHO/WHO Collaborating Centre for Nursing Research Development, Ribeirão Preto, SP, Brazil.

**Keywords:** Nursing, Global Health, Health Personnel, Staff Development, Education, Nursing, Education, Nursing, Continuing, Enfermagem, Saúde Global, Pessoal de Saúde, Desenvolvimento de Pessoal, Educação em Enfermagem, Educação Continuada em Enfermagem, Enfermería, Salud Global, Personal de Salud, Desarrollo de Personal, Educación em Enfermería, Educación Continua em Enfermería

## Abstract

**Objective:**

to understand from the complex perspective the connections established between the Strategic Directions for Strengthening Nursing and Midwifery, delimited by the World Health Organization; to discuss the implications of these strategies for the investment of human resources in nursing and midwifery, with a view to strengthening the technical health capacity to face global health demands.

**Method:**

a documentary research, carried out from official WHO documents, from September to October 2019. A categorical analysis technique was performed, and the interpretation of the data was achieved based on the theoretical framework of Complexity.

**Results:**

three interdependent categories were defined, namely: challenges for the training of human resources in nursing and midwifery in order to meet the needs for global health; challenges for the development of the work of nursing and midwifery professionals in different contexts of practice; challenges for governments, leaders and health services to strengthen human resources in nursing and midwifery.

**Conclusion:**

the strategic directions corroborate the complex perspective because they value multidimensionality in the challenges for the professional practice of nurses and obstetricians. However, these challenges are also related to contextual, political, and leadership factors.

## Abstract (pt)

**Objetivo:**

compreender, a partir da perspectiva complexa, as conexões estabelecidas entre as Orientações Estratégicas para o Fortalecimento da Enfermagem e Obstetrícia, delimitadas pela Organização Mundial da Saúde; discutir as implicações dessas estratégias para o investimento de recursos humanos da enfermagem e obstetrícia, com vistas ao fortalecimento da capacidade técnica de saúde para o enfrentamento das demandas de saúde global.

**Método:**

pesquisa documental, realizada a partir de documentos oficiais da OMS, no período de setembro a outubro de 2019. Foi realizada técnica de análise categorial, sendo a interpretação dos dados alcançada a partir do referencial teórico da Complexidade.

**Resultados:**

foram delimitadas três categorias, interdependentes, a saber: desafios para a formação de recursos humanos em enfermagem e obstetrícia com vistas ao atendimento das necessidades para a saúde global; desafios para o desenvolvimento do trabalho dos profissionais de enfermagem e obstetrícia nos diferentes contextos de atuação; desafios para os governos, lideranças e serviços de saúde para o fortalecimento de recursos humanos em enfermagem e obstetrícia.

**Conclusão:**

as orientações estratégicas corroboram a perspectiva complexa porque valorizam a multidimensionalidade nos desafios para o exercício profissional de enfermeiros e obstetrizes. Todavia, esses desafios estão relacionados, também, aos fatores contextuais, políticos e de liderança.

## Abstract (es)

**Objetivo:**

comprender, desde una perspectiva compleja, las conexiones establecidas entre las Orientaciones Estratégicas para el Fortalecimiento de la Enfermería y la Partería, delimitadas por la Organización Mundial de la Salud; discutir las implicaciones de estas estrategias para la inversión de recursos humanos en enfermería y partería, con miras al fortalecimiento de la capacidad técnica de salud para afrontar la demanda global de servicios de salud.

**Método:**

investigación documental, realizada con base en documentos oficiales de la OMS, de septiembre a octubre de 2019. Se llevó a cabo un análisis categorial y la interpretación de los datos se logró a partir del marco teórico de la Complejidad.

**Resultados:**

se definieron tres categorías interdependientes, a saber: desafíos para la capacitación de recursos humanos en enfermería y partería a fin de satisfacer las necesidades de la salud global; desafíos para el desarrollo del trabajo de los profesionales de enfermería y partería en diferentes contextos de actuación; desafíos para los gobiernos, las direcciones y los servicios de salud para fortalecer los recursos humanos en enfermería y partería.

**Conclusión:**

las orientaciones estratégicas corroboran la perspectiva compleja porque valoran la multidimensionalidad en los desafíos para la práctica profesional de enfermeros y obstetras. No obstante, estos desafíos también se hallan relacionados con factores contextuales, políticos y de liderazgo.

## Introduction

The development of the nations is in line with the complex and dynamic health demands of their peoples, which establish implications in different social, economic, and political contexts for guaranteeing human dignity based on people’s quality of life^(^ 1 ^-^ 3 ^)^.

In this context, it is necessary to consider the importance of human health resources so as to implement and strengthen strategic actions that guarantee efficient health and care practices to people in the face of current and coming challenges for local and global health, estimated from the epidemiology of aging population, chronic health conditions, emerging and neglected diseases, mental health, and social inequities, among others^(^ 4 ^-^ 5 ^)^.

Thus, nursing and midwifery must be understood as professions that, within the scope of human resources for health, assume and practice valuable contributions to ensure access to health services and practices for people and their families in different care contexts^(^ 6 ^-^ 7 ^)^. However, it is essential to understand it beyond the profession itself and its reach, significantly, different leaders, which can result in unfolding actions performed by nursing and midwifery, so that they can reach the understanding that investing in these professionals is equivalent to investing in people’s quality of life, as well as efficient management of resources to reduce expenses with the fight against diseases^(^ 8 ^-^ 9 ^)^.

Furthermore, by guaranteeing decent conditions for the maintenance of the economically active population, based on the health and care actions performed by nursing and midwifery, it is possible to conceive the interdependence relationship between these professionals and the economic development of nations. Therefore, in this relationship, there is the principle of the recursive circuit of complexity, “in which products and effects are themselves producers and causes of what produces them”^(^ 10 ^)^.

On the other hand, it must be considered that the development of the nursing and midwifery work process with a view to universal access to health is conditioned to the political context for the exercise of the autonomy of these professionals, as well as the development of skills to better intervene^(^ 5 ^,^ 11 ^)^. Despite this reality, the connections between macro- and micro-politics, which are positioned in multidimensional perspectives, are fundamental to guarantee strategies for strengthening human resources of nurses and obstetricians, with regard to the commitment of these professionals to consolidated training, as well as of the health institutions to implement dignified conditions so that they act with the necessary autonomy to reach their full potential.

Therefore, the World Health Organization (WHO) launches the set of Strategic Directions for Strengthening Nursing and Midwifery Services^(^ 5 ^,^ 12 ^-^ 13 ^)^ and recently, in partnership with the International Nurses Council, the global program for the valorization of Nursing and Midwifery, *Nursing Now* ^(^ 14 ^)^, with a focus on leadership for care practices that advance new and distinct health scenarios, among other purposes.

On the other hand, it is advocated that these strategies should be perceived in their complexities, that is, interconnected in their contextual, multidimensional and dynamic perspectives. It follows from this logic the ability of these strategies to be conceived as guiding axes for the connections between macro- and micro-politics that involve investment and development for the human resources of health, with a view to expanding conditions of understanding by the formulators of public policies on the importance of nursing and midwifery to the world. Therefore, it is envisaged that civil society, politicians, and health employers appreciate the understanding that investment in these professionals is essential to respond to current and future epidemiological challenges^(^ 5 ^)^.

In this sense, the question is the following: How can the complex perspective favor the necessary understanding of the challenges related to the strengthening of human resources in nursing and midwifery based on the strategic directions defined for the strengthening of these professionals?

Therefore, the objectives of the research were the following: to understand from a complex perspective the connections established between the Strategic Directions for Strengthening Nursing and Midwifery delimited by the WHO; to discuss the implications of these strategies for the investment of human resources in nursing and midwifery, with a view to strengthening the technical health capacity to face the global health demands.

## Method

A documentary research, carried out based on official WHO documents. For the analysis process, the categorical analysis technique was performed and the interpretation of the data was achieved based on the theoretical framework of Complexity, in the perspective of Morin^(^ 10 ^,^ 15 ^)^.

Complex Thinking aims at understanding phenomena from the interactions among the parts that constitute them, in a dynamic, procedural, and non-linear perspective. For that, it uses principles that allow understanding the complexity inserted in the dynamics of operation of the analyzed phenomenon, such as the recursive organizational principle, which considers that a phenomenon is at the same time a product and producer of itself^(^ 10 ^,^ 15 ^)^. In this sense, the strengthening of nursing and midwifery is now understood from internal and external mechanisms to the work process of these professionals.

In the complex perspective, the principles complement each other to better explain the phenomenon in its living dynamics of operation. This way, for example, another principle can be added, namely: autonomy-dependence^(^ 15 ^)^, which allows systems to self-organize their parts for the balance and maintenance of the whole. Other principles for conceiving complexity are approached in the discussion of the results of this research.

The documents used as data sources were the following: Strategic Directions for Strengthening Nursing and Midwifery Services (2002-2008)^(^ 12 ^)^, Strategic Directions for Nursing and Midwifery (2011-2015)^(^ 13 ^)^, Global Strategic Directions for strengthening Nursing and Midwifery (2016-2020)^(^ 5 ^)^.

The analysis of the documents was carried out based on three defining questions for the categorization process, namely: a) What are the challenges for the training of human resources in nursing and midwifery in order to meet the needs for global health? b) What are the challenges for the development of nursing and midwifery work in the different contexts of professional practice? C) How can governments, leaders and health services favor the development of human resources for nursing and midwifery?

The analytical process of the documents based on the questions that led to analysis took place from the transcription of the data in figures with four columns in a *Word* file and compared with each other, grouped by similarities, in order to form explanatory categories of the phenomenon under study. The entire categorization process was carried out based on the complexity framework and analyzed by three independent researchers. Data was collected directly from the official WHO websites, from September to October 2019.

As this is a documentary research with data in the public domain, the research was not submitted to the Human Research Ethics Committee.

## Results

The grouping of the results is presented by categorization in three figures, from the questions that induce analysis. To identify the WHO official documents, the following denomination was used: Directions 2002-2008; Directions 2011-2015; Directions 2016-2017.

It should be highlighted that the strategic directions for strengthening nursing and midwifery, in their first versions, that is, 2002-2008 and 2011-2016, listed the set of spheres of main results, with the respective objectives for each sphere. On the other hand, the 2016-2020 version addressed themes with objectives, strategies, and their contextual relations (global, national, regional, and partnership). However, for the presentation of the results, in this article the analyzed material is presented in actions and related factors for each generated category.

Thus, Figure 1 reveals the connections established between actions and related factors present in the strategic directions for the training of human resources in nursing and midwifery, with a view to meeting the needs for global health.

**Figure 1:** Challenges for the training of human resources in nursing and midwifery with a view to meeting the needs for global health

Strategic Direction 2002-2008	*Action: Education of health personnel for nursing and midwifery services*
	*Related factors:* the dynamics of the health services requires professionals with the necessary competences to meet their demands. The strengthening of professional competences in nursing and midwifery is a result of this need.
Strategic Direction 2011-2016	*Action*: *Nursing and midwifery policy and practice*
	*Related factors:* evidence bases for nursing and midwifery. It aims to build an evidence base for the nursing and midwifery practice by means of research, and to ensure that it is used to change the practice.
Strategic Direction 2011-2016	*Action*: *Education, training and career development*
	*Related factors:* provision of nursing and midwifery workforce. To this end, it aims to ensure pre-service and continuing education programs in all the levels of nursing and midwifery to produce an adequate supply of competent professionals. In addition, it aims to ensure that nursing and midwifery education/training programs are grouped into adequate teaching resources. Also to develop knowledge by means of post-basic education, guidance, and other career development activities.

Figure 2 shows the connections established to face the challenges with a view to the development of the work of nursing and midwifery professionals.

**Figure 2:** Challenges for the development of the work of nursing and midwifery professionals in different work contexts

Strategic Direction 2002-2008	*Action: Management of health personnel for nursing and midwifery services*
	*Related factors*: establishment of national employment policies that seek equity for the gender relations that permeate nursing and midwifery with healthy and safe working conditions, with salary dignity, recognition, and appreciation of the nursing and midwifery competences. Based on the above, in addition to hiring, it aims to retain the staff.
Strategic Direction 2002-2008	*Action: Practice and health system improvement*
	*Related factors*: given the dynamism of the health systems, nursing and midwifery need to increasingly base their decision-making and care/management models based on the best scientific evidence, with a view to the best care practices. From this context, improving access to efficient nursing and midwifery services to the individual, family, and community derives as an objective.
Strategic Direction 2011-2016	*Action: Contribution to the strengthening of health systems and services*
	*Related factors*: contribution to person-centered care. This way, it aims to enable nurses and midwives to play a greater role in ensuring that the design, delivery, and performance of the health systems meet people's needs.
Strategic Direction 2011-2016	*Action: Nursing and midwifery policy and practice*
	*Related factors*: improving the professional position of nursing and midwifery
Strategic Direction 2011-2016	*Action*: *Nursing and midwifery workforce management*
	*Related factors*: performance improvement. Thus, it aims to promote a positive work environment, with support supervision for the ideal performance of the nursing and midwifery workforce.
Strategic Direction 2011-2016	*Action*: *Partnership for nursing and midwifery services*
	*Related factors*: it is necessary to encourage active and systematic collaboration among nursing and midwifery organizations, in addition to community-based organizations, professional groups, and the government. Therefore, it aims at the following: implementation and monitoring of these strategic directions, and articulation and strengthening of effective networks and partnerships to improve nursing and midwifery services.
Strategic Direction 2016-2020	*Action*: *Ensuring an educated, competent and motivated nursing and midwifery workforce within effective and responsive health systems at all levels and in different settings*
	*Related factors*: educating, recruiting, implementing, and maintaining the adequate number of nursing and midwifery workers with the necessary competences, strengthened with resources favorable to the performance of work, governed by professional regulations. To this end, it establishes the following as a strategy: to align investments and to coordinate plans for the development of nursing and midwifery in the management of the workforce.
Strategic Direction 2016-2020	*Action*: *Working together to maximize the capacities and potentials of nurses and midwives through intra- and inter-professional collaborative partnerships, education and continuing professional development*
	*Related factors*: to optimize the impact of nursing and midwifery on the health systems, at all levels of health care, by means of inter- and intra-professional collaboration and partnership. For that, it is necessary to outline, monitor, and evaluate roles/responsibilities of the nursing and midwifery workforce to promote education and collaborative practice.

Figure 3 shows the results regarding the challenges that must be assumed by the governments, leaders, and health services to strengthen human resources in nursing and midwifery.

**Figure 3:** Challenges for the governments, leaders, and health services to strengthen human resources in nursing and midwifery

Strategic Direction 2002-2008	*Action: Health planning, advocacy and political commitment*
	*Related factors*: provision of counseling of the nursing and midwifery services adequate to the nation's health and development plans. Three objectives are defined: strengthening the mechanisms for planning and intervention in the field of human resources policy, in order to contribute to the efficient work process of nursing and midwifery; mobilization of different actors (normative bodies, civil society, and professionals, among others) to promote mechanisms that allow for the strengthening of nursing and midwifery services; political and leadership participation in all scopes.
Strategic Direction 2002-2008	*Action: Stewardship and governance*
	*Related factors*: the administration and governance of the nursing and midwifery services comprise government, civil society, and health professionals committed to the quality of people's health care. From the above, two objectives are listed: advising governments on the development of administration and governance of healthy health systems, with a focus on nursing and midwifery services; granting and authorizing nursing and midwifery professionals to their regulatory bodies to assume the responsibility for self-regulation and the quality of care.
Strategic Direction 2011-2016	*Action: Contribution to the strengthening of health systems and services*
	*Related factors*: leadership for health. It aims to train nurses and midwives to lead at all levels of care in the health system.
Strategic Direction	*Action*: *Nursing and midwifery policy and practice*
	*Related factors*: to ensure that nursing and midwifery policies are an integral part of the overall formulation of health policies.
Strategic Direction 2011-2016	*Action: Nursing and midwifery workforce management*
	*Related factors*: to ensure that the national development plans include appropriate health strategies and to the nursing and midwifery services.
Strategic Direction 2011-2016	*Action*: *Partnership for nursing and midwifery services*
	*Related factors*: administration and governance - in order to help governments support the strengthening of solid administration and governance, especially in nursing and midwifery services.
Strategic Direction 2016-2020	*Action: Optimizing policy development, effective leadership, management and governance*
	*Related factors:* entrance and active participation of nursing and midwifery leaders at all levels of policy formulation, planning, and program formulation, including the generation of evidence for decision-making. Therefore, it is necessary to prepare nursing leaders for the current health challenges, with the necessary competence, including in the formulation of policies, and in knowledge management based on evidence for the efficient work process.
Strategic Direction 2016-2020	*Action: Mobilizing political will to invest in building effective evidence-based nursing and midwifery workforce development*
	*Related factors*: to establish structures that allow nurses and midwives to be empowered, in order to achieve effective engagement and contribute to the development of health policies, so as to increase the quantity and quality of the health services. To this end, political support from the health systems and civil society must be built to ensure that the policies created to achieve universal health coverage and the Sustainable Development Goals involve people-centered nursing and midwifery services.

## Discussion

For Complex Thinking, strategy means a mechanism to better deal with the objective reality, because reality itself is permeated by uncertainties, risks, and illusions that may not be controlled in its entirety by the action mechanisms defined by man^(^ 15 ^)^. However, it is up to the strategies to predict better conditions to deal with the dynamic phenomena of reality^(^ 16 ^)^.

Therefore, in the health context in which nursing and midwifery play important roles^(^ 5 ^)^, the strategies for strengthening them must contemplate the possibilities to intervene based on current and future challenges. For this reason, the strategic directions, especially their most recent version^(^ 5 ^)^, are supported by a contextual perspective in a global logic, based on evidence that calls for the strengthening of these professionals to exercise their competences.

The following documents emerge from this reality: The WHO 2013 directions to transform and expand the education and training of the health professionals, namely: the World Midwifery Study Report^(^ 17 ^)^; the 2013-2020 Mental Health Action Plan^(^ 18 ^)^; the 2016-2030 Global Strategy for the Health of Women, Children and Adolescents^(^ 19 ^)^; the Strategy to End Preventable Maternal Mortality; and the World Report on Aging and Health^(^ 20 ^)^, among others.

For all these issues, nursing and midwifery perform fundamental actions to effectively develop these strategies and recommendations for global health. Thus, as highlighted by Complex Thinking in signaling the contextual importance of interactions established for the development of a given phenomenon, the strategic directions for strengthening nursing and midwifery were throughout its editions, reinforcing the importance of the context for the efficient planning and execution of strategies to strengthen these professionals.

From this perspective, the three categories highlight the relevance of the context for the implementation of the necessary actions adopted by the WHO Member States to implement their directions for coverage and universal access to health, for example^(^ 21 ^)^. Thus, the development of professional competences, the improvement of the working conditions, and the encouragement of leaders, governments, and institutions for nursing and midwifery conceive the contextual perspective as indispensable to the full reach of the strategic directions.

It is in the work context that nurses and midwives face challenges of cultural origin, codified in power relations that imply limitations for the full development of these professionals. From the above, the strategic directions have advanced towards the signs referring to the exercise of leadership, professional training, and occupation of decision-making spaces so that nursing and midwifery can increasingly achieve their goals with the different groups with which they work.

Nevertheless, the contextual interactions are necessary for the production of efficient care. For this reason, the focus of complex thinking is the ability to know reality in a multifaceted logic of knowledge and actions^(^ 15 ^)^. From this reality, the valorization of inter-disciplinarity and inter-sectoriality can be seen, as highlighted by the most current version of the strategic directions.

Although in several countries nursing and midwifery make up more than 50% of the human health resources, the work process of these professionals is articulated in a broader objective, which requires a multidimensional approach based on the interdisciplinary strategy in the context of health. With this, it can be revealed that two dimensions are approached in a dialogical perspective which complement each other, namely: professional autonomy for nursing and midwifery and, at the same time, better integration with the relational processes that configure the driving force of health care of the multidisciplinary team.

Furthermore, it is in this set of interactions that another principle of complexity is processed that may contribute to better understand the multifaceted perspective of the strategic directions for strengthening nursing and midwifery and, therefore, the very challenges of these professionals to meet the health and care demands of the people.

This principle is the *unitas-multiplex*, which asks to consider that the whole can be more or less than the sum of its parts^(^ 15 ^)^. This is because the quality of the interactions matters more than their existence because, in a context unfavorable for the development of the parts, the negative interactions of the work process can limit, reduce, and disfavor the potential for transformation of the subject. The opposite can also be revealed by the same principle, that is: the quality of the interactions can enhance strengths of the work nature capable of favoring the nursing and midwifery professionals’ conditions to exercise their roles with excellence, as indicated by the WHO strategic directions.

This research presents as a limitation, in the epistemological order of apprehension of the investigated object, the incapacity of abstractions that can deepen the reality perceived by the professional nurses and midwives about the results presented. This way, other methodologies that address field research, for example, may deepen knowledge about the complex nature of strategic directions for strengthening nursing and midwifery.

On the other hand, the analysis of data from official WHO documents for the strengthening of human resources in nursing and midwifery from the complex perspective revealed the importance of an epistemological approach that allows contextualizing and interconnecting the multiple dimensions involved in the challenges for the strengthening of these professionals, who are permeated by cultural and power structures rooted in decision-making spaces. These realities result in the need for better connections between nursing and midwifery, decent working conditions, public policies, leadership, and global health.

## Conclusion

The strategic directions for strengthening nursing and midwifery corroborate the perspective of complexity, while they value the multidimensionality inserted in the challenges for the professional practice of nurses and midwives.

In this logic, they reinforce the need for connections based on partnerships; the contextual implications for successfully implementing action-interaction strategies; and the interdisciplinary perspective, without, however, devaluing the need for professional autonomy.
